# Diabetes stigma and underdiagnosis: time to change the narrative in the Western Pacific region

**DOI:** 10.1016/j.lanwpc.2025.101764

**Published:** 2025-11-28

**Authors:** The Lancet Regional Health – Western Pacific

Wellbeing, particularly in the workplace, is at the heart of Word Diabetes Day 2025. Approximately 70% of people with diabetes are of working age and face challenges like diabetes stigma which can contribute to feelings of shame, social exclusion, and potential delays in health-care seeking and diagnosis.

According to the latest GBD study (2023), an estimated 248 million people worldwide have undiagnosed diabetes, with the highest prevalence among middle-aged groups (from about 40 to 65 years old), many of whom form a significant portion of the workforce. Globally, diagnosis rates are lowest among young adults (around 15–24 years old), with an estimated 44.2% of people aged 15 years and older with undiagnosed diabetes in 2023. Alarmingly, approximately 50% of adults with diabetes in the Western Pacific region are undiagnosed, making it the region with the second-highest rate of undiagnosed diabetes among all International Diabetes Federation regions. Southeast Asia, East Asia, and Oceania had approximately 81,500 undiagnosed cases per 1000 people, reflecting a 40.4% increase from 2000 to 2023. By contrast, high-income Asia–Pacific countries had a much lower rate of undiagnosed diabetes (4770 cases per 1000 people), with a 4.5% decrease over the same period. Delayed diagnosis can lead to serious health consequences, including increased risks of cardiovascular disease, kidney damage, and lower limb amputations.

Factors contributing to delayed health-care seeking include the often-asymptomatic nature of diabetes, limited availability and access to screening programmes, and potentially lower uptake. Suboptimal cut-offs in widely used oral glucose tolerance tests for Asian populations might also hinder early detection. Additionally, diabetes stigma might discourage individuals from seeking medical care. Recent studies suggest that cultural values like collectivism and familism central to many Western Pacific cultures shape how people with diabetes experience and manage stigma. For example, a nationwide study in Singapore found that although only 19% of people believed individuals are responsible for their condition, a larger proportion were unwilling to have someone with diabetes marry into their family (28%), believed that people with diabetes used more health care than average (69%), and thought it affected health insurance (81%). These beliefs are likely linked to cultural norms around participation in family events and community responsibilities. Similarly, in Pacific Islander and Māori communities, stigma related to diabetes and medication use is associated with feelings of judgement or blame, which can hinder seeking family support, an important aspect of their cultural identity and health.

Raising awareness about perceptions of diabetes across different cultures is crucial for reducing underdiagnosis and stigma, and for improving prevention and care. During the 16th Scientific Meeting of the Asian Association for the Study of Diabetes, consensus was reached on the need to revise stigmatising diabetes terminology across Asia, with plans to monitor progress and continue discussions. Professor Kadowaki, Chair of the International Diabetes Federation Western Pacific Region at the time, highlighted that in some countries such as Japan, China, and South Korea, terms for diabetes often include sugar and urine, which can cause shame and reinforce harmful stereotypes, such as diabetes being a consequence of laziness or poor lifestyle choices. For example, in Japan, the current term (tō-nyō-byō) translates to “sugar urine disease”. A new Japanese term in Katakana (“ダイアベティス”) has been proposed to align with academic standards and international guidelines, aiming to help reduce stigma. In Hong Kong, the Young Onset Diabetes Care Community Campaign seeks to increase public awareness of the importance of early diagnosis of young-onset diabetes and to empower young people as ambassadors of diabetes care.

Ongoing advocacy efforts must involve all stakeholders, including people with diabetes, their families, and health-care professionals, and prioritise a dialogue-driven approach that employs culturally sensitive, stigma-free, and empowering language. Diabetes is a medical condition that could affect anyone, anywhere. A combination of genetics, age, and other health conditions contribute to diabetes risk. Laziness is not a cause of diabetes. With early diagnosis, high-quality care, and strong family and community support, many people with diabetes can lead active, healthy, and fulfilling lives. To effectively address diabetes stigma and underdiagnosis, we must change how we think and speak about diabetes as a community. We must harness the region's strengths, such as collectivism and familism, to develop culturally tailored interventions and community-based approaches that involve family and community members, to reduce stigma and improve prevention and care.

In collaboration with the International Diabetes Federation—Western Pacific Congress, jointly held with the Australasian Diabetes Congress (ADC) and Metabolic Diseases 2026, we invite original research submissions on innovative strategies to enhance diabetes prevention, diagnosis, management, and stigma reduction in the region. Together, we can drive meaningful changes and build a more informed, inclusive, and supportive community for everyone living with diabetes in the Western Pacific.

